# Supplementary material for: Iterative improvement in the automatic modular design of robot swarms
Source: PeerJ Comput Sci. 2020 Dec 7;6:e322. doi: 10.7717/peerj-cs.322 (PMC7924708; doi:10.7717/peerj-cs.322)
Supplement: Supplemental Information 3 [file peerj-cs-06-322-s003.zip › argos3/doc/api/standalone/a00598_source.html]

ARGoS: plugins/simulator/media/rab\_medium.cpp Source File


- Main Page
- Related Pages
- Namespaces
- Classes
- Files

- File List
- File Members

# plugins/simulator/media/rab\_medium.cpp

Go to the documentation of this file.

```
00001 #include "rab_medium.h"
00002 #include <argos3/core/simulator/entity/embodied_entity.h>
00003 #include <argos3/core/simulator/simulator.h>
00004 #include <argos3/core/simulator/space/space.h>
00005 #include <argos3/core/simulator/space/positional_indices/grid.h>
00006 #include <argos3/core/utility/configuration/argos_exception.h>
00007 #include <argos3/core/utility/logging/argos_log.h>
00008 
00009 namespace argos {
00010 
00011    /****************************************/
00012    /****************************************/
00013 
00014    CRABMedium::CRABMedium() {
00015    }
00016 
00017    /****************************************/
00018    /****************************************/
00019 
00020    CRABMedium::~CRABMedium() {
00021    }
00022 
00023    /****************************************/
00024    /****************************************/
00025 
00026    void CRABMedium::Init(TConfigurationNode& t_tree) {
00027       try {
00028          CMedium::Init(t_tree);
00029          /* Get the positional index method */
00030          std::string strPosIndexMethod("grid");
00031          GetNodeAttributeOrDefault(t_tree, "index", strPosIndexMethod, strPosIndexMethod);
00032          /* Get the arena center and size */
00033          CVector3 cArenaCenter;
00034          CVector3 cArenaSize;
00035          TConfigurationNode& tArena = GetNode(CSimulator::GetInstance().GetConfigurationRoot(), "arena");
00036          GetNodeAttribute(tArena, "size", cArenaSize);
00037          GetNodeAttributeOrDefault(tArena, "center", cArenaCenter, cArenaCenter);
00038          /* Create the positional index for embodied entities */
00039          if(strPosIndexMethod == "grid") {
00040             size_t punGridSize[3];
00041             if(!NodeAttributeExists(t_tree, "grid_size")) {
00042                punGridSize[0] = cArenaSize.GetX();
00043                punGridSize[1] = cArenaSize.GetY();
00044                punGridSize[2] = cArenaSize.GetZ();
00045             }
00046             else {
00047                std::string strPosGridSize;
00048                GetNodeAttribute(t_tree, "grid_size", strPosGridSize);
00049                ParseValues<size_t>(strPosGridSize, 3, punGridSize, ',');
00050             }
00051             CGrid<CRABEquippedEntity>* pcGrid = new CGrid<CRABEquippedEntity>(
00052                cArenaCenter - cArenaSize * 0.5f, cArenaCenter + cArenaSize * 0.5f,
00053                punGridSize[0], punGridSize[1], punGridSize[2]);
00054             m_pcRABEquippedEntityGridUpdateOperation = new CRABEquippedEntityGridEntityUpdater(*pcGrid);
00055             pcGrid->SetUpdateEntityOperation(m_pcRABEquippedEntityGridUpdateOperation);
00056             m_pcRABEquippedEntityIndex = pcGrid;
00057          }
00058          else {
00059             THROW_ARGOSEXCEPTION("Unknown method \"" << strPosIndexMethod << "\" for the positional index.");
00060          }
00061       }
00062       catch(CARGoSException& ex) {
00063          THROW_ARGOSEXCEPTION_NESTED("Error in initialization of the range-and-bearing medium", ex);
00064       }
00065    }
00066 
00067    /****************************************/
00068    /****************************************/
00069 
00070    void CRABMedium::PostSpaceInit() {
00071       Update();
00072    }
00073 
00074    /****************************************/
00075    /****************************************/
00076 
00077    void CRABMedium::Reset() {
00078       /* Reset positional index of RAB entities */
00079       m_pcRABEquippedEntityIndex->Reset();
00080       /* Delete routing table */
00081       for(TRoutingTable::iterator it = m_tRoutingTable.begin();
00082           it != m_tRoutingTable.end();
00083           ++it) {
00084          it->second.clear();
00085       }
00086    }
00087 
00088    /****************************************/
00089    /****************************************/
00090 
00091    void CRABMedium::Destroy() {
00092       delete m_pcRABEquippedEntityIndex;
00093       if(m_pcRABEquippedEntityGridUpdateOperation != NULL) {
00094          delete m_pcRABEquippedEntityGridUpdateOperation;
00095       }
00096    }
00097 
00098    /****************************************/
00099    /****************************************/
00100 
00101    static UInt64 HashRABPair(const std::pair<CRABEquippedEntity*, CRABEquippedEntity*>& c_pair) {
00102       UInt64 unA = *reinterpret_cast<unsigned long long*>(c_pair.first) & 0xFFFFFFFF;
00103       UInt64 unB = *reinterpret_cast<unsigned long long*>(c_pair.second) & 0xFFFFFFFF;
00104       return (unA << 32) | unB;
00105    }
00106 
00107    void CRABMedium::Update() {
00108       /* Update positional index of RAB entities */
00109       m_pcRABEquippedEntityIndex->Update();
00110       /* Delete routing table */
00111       for(TRoutingTable::iterator it = m_tRoutingTable.begin();
00112           it != m_tRoutingTable.end();
00113           ++it) {
00114          it->second.clear();
00115       }
00116       /* This map contains the pairs that have already been checked */
00117       std::map<UInt64, std::pair<CRABEquippedEntity*, CRABEquippedEntity*> > mapPairsAlreadyChecked;
00118       /* Iterator for the above structure */
00119       std::map<UInt64, std::pair<CRABEquippedEntity*, CRABEquippedEntity*> >::iterator itPair;
00120       /* Used as test key */
00121       std::pair<CRABEquippedEntity*, CRABEquippedEntity*> cTestKey;
00122       /* Used as hash for the test key */
00123       UInt64 unTestHash;
00124       /* The ray to use for occlusion checking */
00125       CRay3 cOcclusionCheckRay;
00126       /* Buffer for the communicating entities */
00127       CSet<CRABEquippedEntity*> cOtherRABs;
00128       /* Buffer to store the intersection data */
00129       SEmbodiedEntityIntersectionItem sIntersectionItem;
00130       /* The distance between two RABs in line of sight */
00131       Real fDistance;
00132       /* Go through the RAB entities */
00133       for(TRoutingTable::iterator it = m_tRoutingTable.begin();
00134           it != m_tRoutingTable.end();
00135           ++it) {
00136          /* Get a reference to the current RAB entity */
00137          CRABEquippedEntity& cRAB = *(it->first);
00138          /* Initialize the occlusion check ray start to the position of the robot */
00139          cOcclusionCheckRay.SetStart(cRAB.GetPosition());
00140          /* For each RAB entity, get the list of RAB entities in range */
00141          cOtherRABs.clear();
00142          m_pcRABEquippedEntityIndex->GetEntitiesAt(cOtherRABs, cRAB.GetPosition());
00143          /* Go through the RAB entities in range */
00144          for(CSet<CRABEquippedEntity*>::iterator it2 = cOtherRABs.begin();
00145              it2 != cOtherRABs.end();
00146              ++it2) {
00147             /* Get a reference to the RAB entity */
00148             CRABEquippedEntity& cOtherRAB = **it2;
00149             /* First, make sure the entities are not the same */
00150             if(&cRAB != &cOtherRAB) {
00151                /* Proceed if the pair has not been checked already */
00152                if(&cRAB < &cOtherRAB) {
00153                   cTestKey.first = &cRAB;
00154                   cTestKey.second = &cOtherRAB;
00155                }
00156                else {
00157                   cTestKey.first = &cOtherRAB;
00158                   cTestKey.second = &cRAB;
00159                }
00160                unTestHash = HashRABPair(cTestKey);
00161                itPair = mapPairsAlreadyChecked.find(unTestHash);
00162                if(itPair == mapPairsAlreadyChecked.end() ||   /* Pair does not exist */
00163                   itPair->second.first != cTestKey.first ||   /* Pair exists, but first RAB involved is different */
00164                   itPair->second.second != cTestKey.second) { /* Pair exists, but second RAB involved is different */
00165                   /* Mark this pair as already checked */
00166                   mapPairsAlreadyChecked[unTestHash] = cTestKey;
00167                   /* Proceed if the message size is compatible */
00168                   if(cRAB.GetMsgSize() == cOtherRAB.GetMsgSize()) {
00169                      /* Proceed if the two entities are not obstructed by another object */
00170                      cOcclusionCheckRay.SetEnd(cOtherRAB.GetPosition());
00171                      if((!GetClosestEmbodiedEntityIntersectedByRay(sIntersectionItem,
00172                                                                    cOcclusionCheckRay,
00173                                                                    cRAB.GetEntityBody())) ||
00174                         (&cOtherRAB.GetEntityBody() == sIntersectionItem.IntersectedEntity)) {
00175                         /* If we get here, the two RAB entities are in direct line of sight */
00176                         /* cRAB can receive cOtherRAB's message if it is in range, and viceversa */
00177                         /* Calculate square distance */
00178                         fDistance = cOcclusionCheckRay.GetLength();
00179                         if(fDistance < cOtherRAB.GetRange()) {
00180                            /* cRAB receives cOtherRAB's message */
00181                            it->second.insert(&cOtherRAB);
00182                         }
00183                         if(fDistance < cRAB.GetRange()) {
00184                            /* cOtherRAB receives cRAB's message */
00185                            m_tRoutingTable[&cOtherRAB].insert(&cRAB);
00186                         }
00187                      } // occlusion found?
00188                   } // is msg size the same?
00189                } // is check necessary?
00190             } // are entities the same?
00191          } // for entities in range
00192       } // for routing table
00193    }
00194 
00195    /****************************************/
00196    /****************************************/
00197 
00198    void CRABMedium::AddEntity(CRABEquippedEntity& c_entity) {
00199       m_tRoutingTable.insert(
00200          std::make_pair<CRABEquippedEntity*, CSet<CRABEquippedEntity*> >(
00201             &c_entity, CSet<CRABEquippedEntity*>()));
00202       m_pcRABEquippedEntityIndex->AddEntity(c_entity);
00203    }
00204 
00205    /****************************************/
00206    /****************************************/
00207 
00208    void CRABMedium::RemoveEntity(CRABEquippedEntity& c_entity) {
00209       TRoutingTable::iterator it = m_tRoutingTable.find(&c_entity);
00210       if(it != m_tRoutingTable.end()) {
00211          m_pcRABEquippedEntityIndex->RemoveEntity(c_entity);
00212          m_tRoutingTable.erase(it);
00213       }
00214       else {
00215          THROW_ARGOSEXCEPTION("Can't erase entity \"" << c_entity.GetId() << "\" from RAB medium \"" << GetId() << "\"");
00216       }
00217    }
00218 
00219    /****************************************/
00220    /****************************************/
00221 
00222    const CSet<CRABEquippedEntity*>& CRABMedium::GetRABsCommunicatingWith(CRABEquippedEntity& c_entity) const {
00223       TRoutingTable::const_iterator it = m_tRoutingTable.find(&c_entity);
00224       if(it != m_tRoutingTable.end()) {
00225          return it->second;
00226       }
00227       else {
00228          THROW_ARGOSEXCEPTION("RAB entity \"" << c_entity.GetId() << "\" is not managed by the RAB medium \"" << GetId() << "\"");
00229       }
00230    }
00231 
00232    /****************************************/
00233    /****************************************/
00234 
00235    REGISTER_MEDIUM(CRABMedium,
00236                    "range_and_bearing",
00237                    "Carlo Pinciroli [ilpincy@gmail.com]",
00238                    "1.0",
00239                    "It simulates the communication across range-and-bearing-equipped robots.",
00240                    "This medium is required to simulate communication across range-and-bearing-\n"
00241                    "equipped robots. You need to add it to the <media> section every time you add\n"
00242                    "a range-and-bearing-equipped entity whose controller has a range-and-bearing\n"
00243                    "device activated.\n\n"
00244                    "REQUIRED XML CONFIGURATION\n\n"
00245                    "<range_and_bearing id=\"rab\" />\n\n"
00246                    "OPTIONAL XML CONFIGURATION\n\n"
00247                    "None for the time being\n",
00248                    "Under development"
00249       );
00250 
00251    /****************************************/
00252    /****************************************/
00253 
00254 }
```

---

Generated on 10 Jul 2018 for ARGoS by 
 1.6.1
